# Red Fluorescent Protein-Aequorin Fusions as Improved Bioluminescent Ca^2+^ Reporters in Single Cells and Mice

**DOI:** 10.1371/journal.pone.0019520

**Published:** 2011-05-11

**Authors:** Adil Bakayan, Cecilia F. Vaquero, Fernando Picazo, Juan Llopis

**Affiliations:** Facultad de Medicina and Centro Regional de Investigaciones Biomédicas (CRIB), University of Castilla-La Mancha, Albacete, Spain; University of California, Berkeley, United States of America

## Abstract

Bioluminescence recording of Ca^2+^ signals with the photoprotein aequorin does not require radiative energy input and can be measured with a low background and good temporal resolution. Shifting aequorin emission to longer wavelengths occurs naturally in the jellyfish *Aequorea victoria* by bioluminescence resonance energy transfer (BRET) to the green fluorescent protein (GFP). This process has been reproduced in the molecular fusions GFP-aequorin and monomeric red fluorescent protein (mRFP)-aequorin, but the latter showed limited transfer efficiency. Fusions with strong red emission would facilitate the simultaneous imaging of Ca^2+^ in various cell compartments. In addition, they would also serve to monitor Ca^2+^ in living organisms since red light is able to cross animal tissues with less scattering. In this study, aequorin was fused to orange and various red fluorescent proteins to identify the best acceptor in red emission bands. Tandem-dimer Tomato-aequorin (tdTA) showed the highest BRET efficiency (largest energy transfer critical distance *R_0_*) and percentage of counts in the red band of all the fusions studied. In addition, red fluorophore maturation of tdTA within cells was faster than that of other fusions. Light output was sufficient to image ATP-induced Ca^2+^ oscillations in single HeLa cells expressing tdTA. Ca^2+^ rises caused by depolarization of mouse neuronal cells in primary culture were also recorded, and changes in fine neuronal projections were spatially resolved. Finally, it was also possible to visualize the Ca^2+^ activity of HeLa cells injected subcutaneously into mice, and Ca^2+^ signals after depositing recombinant tdTA in muscle or the peritoneal cavity. Here we report that tdTA is the brightest red bioluminescent Ca^2+^ sensor reported to date and is, therefore, a promising probe to study Ca^2+^ dynamics in whole organisms or tissues expressing the transgene.

## Introduction

The biochemical mechanisms involved in Ca^2+^ regulation of a large number of physiological processes have been elucidated [1], [2], [3] in addition to other methods with the help of the Ca^2+^-sensitive photoprotein aequorin [4] and synthetic fluorescent probes [5]. In the early days, luminometry and fluorometry provided a good time resolution at the cost of spatial information, but have since been superseded by imaging techniques given the development of sensitive detectors on the one hand and a large palette of fluorescent proteins (FPs) on the other [6]. The latter has allowed the development of fluorescent recombinant Ca^2+^ indicators by fusing FPs and various Ca^2+^-binding proteins [7].

Aequorin consists of an apoprotein (189 amino acids), a noncovalently-bound chromophoric unit (coelenterazine) and molecular oxygen (as a hydroperoxide of coelenterazine). Binding of Ca^2+^ ions to two or three aequorin EF-hands causes the photoprotein to undergo a conformational change, resulting in the oxidation of coelenterazine, which is converted into coelenteramide with the release of CO_2_ and a flash of blue light (465 nm) [8]. Aequorin-based methods offer the following features: (i) bioluminescence does not require excitation light, thus avoiding problems like phototoxicity, photobleaching and autofluorescence, and making it minimally-invasive with high signal/noise; (ii) availability of coelenterazines with different Ca^2+^-affinities [9] and mutated low Ca^2+^-affinity aequorin [10] allow measuring [Ca^2+^] from 10^−7^ to 10^−3^ M; (iii) aequorin is almost insensitive to changes in pH; (iv) it can be molecularly targeted to different subcellular compartments to report local [Ca^2+^] [10], [11]; (v) it is not present in mammalian cells, and it showed little, if any, toxicity during development in transgenic mice [12]; (vi) aequorin signals are difficult to image because of a combination of low emission quantum yield and low protein stability [13]; each molecule only performs one emission cycle upon Ca^2+^ binding (recharging with the cofactor is relatively slow) [14].

In the jellyfish *Aequorea victoria* from which aequorin was first isolated, the protein is associated in the luminiferous organs with the GFP [15]. They are found at a high concentration with intermolecular distances that allow radiationless energy transfer from the activated oxyluciferin to GFP in a process known as bioluminescence resonance energy transfer (BRET) [16]. This natural phenomenon was mimicked in the laboratory by the molecular fusion of GFP and aequorin (GA) [13], [17]; this fusion showed an increased light-emitting activity within the cytoplasm because of enhanced protein stability and, possibly, quantum yield compared to aequorin alone. Moreover, GA is a bifunctional hybrid in which the expression can be followed by fluorescence of GFP, while the aequorin moiety is the Ca^2+^ sensor. We previously reported the fusion of red fluorescent protein mRFP1.2 and aequorin (mRA) [18], not spectrally identical to mRFP1-aequorin (RA) described by Curie et al [19]. Energy transfer in mRA was limited, but sufficient to allow simultaneous Ca^2+^ measurement in two different organelles by co-expressing appropriately targeted GA and mRA in mammalian cells using a two-channel luminometer [18]. A more efficient energy transfer from aequorin to orange/red FPs would be welcome for this application, and would also allow imaging Ca^2+^ dynamics in live organisms, which has already been accomplished with GA [12]. Since blue/green light is strongly scattered and absorbed by biological tissues [20], [21], developing efficient red Ca^2+^ reporter variants would facilitate measurements in animals.

In this study, we tested three new potential acceptors for aequorin (*mOrange*, and the red FPs *TagRFP* and *tdTomato*) [22], [23]. These molecular fusions were compared with the previously reported mRA [18]. As high energy transfer controls, we used GA and newly-made yellow FP *citrine-aequorin* fusion; all the BRET pairs were compared with aequorin alone. Emission was measured in four spectral bands by imaging the chimeras in live HeLa cells. We achieved an efficient energy transfer between aequorin and the red FP tdTomato, and we further applied this reporter to image Ca^2+^ in single HeLa cells and neurons in primary culture.

## Results

### New Fluorescent Protein-Aequorin Fusions

**Figure 1:**
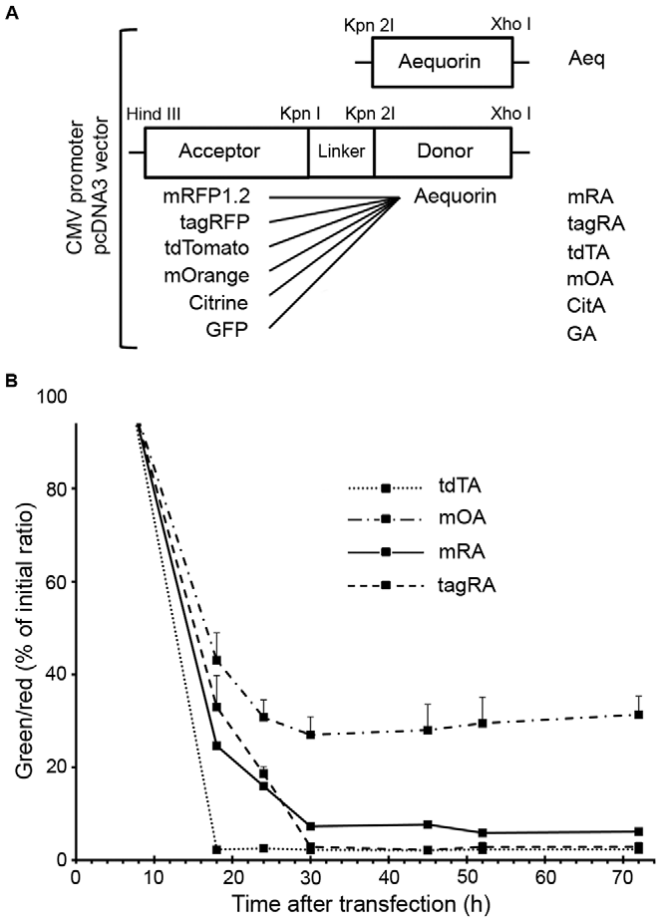
Construction of BRET chimeras and characterization of their red fluorophore maturation. (**A**) Schematic maps and abbreviations of aequorin alone and BRET chimeras for mammalian expression in the pcDNA3 vector. A linker of 17 amino acids between aequorin and fluorescent proteins (FPs) was used in all the chimeras. (**B**) Monitoring red fluorophore maturation of FP-aequorin fusions in HeLa cells. The starting green (excitation 500 nm, emission 542 nm) to red (excitation 550 nm, emission 595 nm) ratio was measured 8 hours after transfection. The green to red ratio (expressed as % of initial ratio) is shown at different times after transfection to estimate the accumulation of the mature orange/red form of the fluorochrome in the fusion proteins. Part of the mOrange mature emission is collected by the green filter. Each point is the mean ± sd of 5 to 26 cells.

Various orange and red FPs were tested as acceptors for energy transfer from donor aequorin. Six different FP fusions with aequorin, plus aequorin alone (Aeq), were compared by expression in mammalian cells. GA [13] and mRA [18] have been previously reported. Four other FPs, citrine [24], mOrange [23] tandem dimer Tomato (tdTomato) [23] and TagRFP [22], were substituted for GFP in the GA construct to yield chimeras CitA, mOA, tdTA and tagRA, respectively (Figure 1A). The same 17-amino acid flexible linker was conserved in all the BRET constructs and aequorin C-terminal sequence was kept intact [25]. The hybrid proteins did not carry targeting signals, therefore transfection in HeLa cells or neurons in culture resulted in a uniform fluorescence in both the cytoplasm and the nucleus with no sign of aggregation or toxicity, as shown with other FP-aequorin fusions [13], [18], [19]. Those cells expressing tdTA showed brighter fluorescence than those producing the other chimeras due to tdTomato's high molar extinction coefficient (138,000 M^−1^ cm^−1^). Three of the fusion proteins were subcloned in bacterial expression vectors (r-Aeq, r-CitA and r-tdTA, renamed with prefix *r-*). When recombinant protein was expressed in *E. coli*, the total amount of purified r-Aeq was almost four times less than that of the r-CitA or r-tdTA fusions. This is consistent with the finding that apoaequorin alone is unstable within the cytosol of mammalian cells with a half-life of approximately 20 min [26], whereas fluorescent proteins are very robust and probably stabilize and protect apoaequorin in the hybrid constructs [13].

### Red Fluorophore Maturation

Red FPs derived from DsRed were believed to pass through a green intermediate stage during red fluorophore formation [27]. More recent evidence points to a branched pathway in which a GFP–like chromophore is a ‘dead-end’ product and the red chromophore proceeds though a blue intermediate, formed before desaturation of the tyrosine Cα–Cβ bond [28], [29] (recently revised in [30]). All the orange and red FPs used in this study (mOrange, tdTomato, TagRFP and mRFP1.2) produced green fluorescence soon after transfection, and strong red fluorescence appeared in all of them but at different maturation rate [23]. Consequently, since the green chromophore overlaps with aequorin emission, the state of red chromophore formation of FP-aequorin chimeras in the cell would influence the final bioluminescence spectrum. The contribution of the GFP-like species in cells at different times after transfection compared to the mature orange/red chromophore was quantified as the ratio of green emission (excitation 500 nm, emission filter 542/27 nm) to red emission (excitation 550 nm, emission filter 595/40 nm). The time dependence of the green/red emission of the FP in chimeras mOA, tdTA, tagRA and mRA in HeLa cells is depicted in Figure 1B. Maturation of tdTomato (large excess of red fluorochrome compared to ‘dead-end’ green form) was essentially complete 18 hours after transfection, whereas that of mOrange, TagRFP and mRFP1.2 in their respective fusions was about 12 hours slower. It should be noted that part of the mOrange mature chromophore fluorescence was transmitted through the 542/27 nm filter used for green species. Therefore, mOA green/red emission ratio stabilized with time as expected, indicating complete fluorophore maturation at about 30 hours, but did not reach low values when compared with the other chimeras. Based on these results, we considered 30 hours post-transfection the minimum time to undertake BRET studies, except for tdTA, which could be used after 18 hours.

### Characterization of the Emission Profile of FP-Aequorin Fusions

If there was an energy transfer between aequorin and the FP in the molecular fusions, the spectral profile of the former would change. Emission of the BRET constructs was measured in four spectral bands in transfected HeLa cells. The imaging setup consisted of an inverted microscope, an emission filterwheel and an EM-CCD camera, all placed under a lightproof cover (Figure S1B); emission filters spanned 200 nm (from 464 nm to 665 nm) with two minor gaps (Figure S2A). Counts were normalized for filter bandwidth and transmission, as well as for camera sensitivity; we refer to each channel (481, 535, 595 and 640) as its center wavelength in nm. The quantification method was validated by reconstructing well-known normalized emission spectra by means of the four channel measurements and corrections. The predicted spectral distribution of aequorin (luminescence) and GFP (fluorescence) (Figure S2B) matched their experimental emission into the four imaging channels (as explained in *Materials and Methods*) (Table 1).

**Table 1:** Validation of the four-channel approach.

		Signal contribution in each emission filter (% of total)			
		481 nm	535 nm	595 nm	640 nm
GFP	Predicted	19.8	71.5	7.4	1.4
	Experimental	20.4±2.4	68.0±2.0	8.7±1.0	2.9±0.7
Aeq	Predicted	66.4	28.8	4.3	0.5
	Experimental	64.1±2.4	27.1±2.0	6.5±1.4	2.4±0.6

Predicted signal contribution in each channel was calculated from the integral of filter transmittance and the known published emission spectra of GFP and luminescence of Aeq (see *Materials and Methods*). The experimental data for GFP were obtained from transfected HeLa cells with off-peak excitation at 420 nm and recording fluorescence signal in the four emission channels, whereas the Aeq data represent bioluminescence contribution during a Ca^2+^ response. Raw counts were corrected for filter bandwidth/transmittance and camera sensitivity (manufactureŕs data); the counts in each channel were divided by the sum of the counts in the four channels, and were expressed as a percentage. The mean±S.E. of 24 cells is shown.

Ca^2+^-dependent luminescence of the BRET constructs was imaged in HeLa cells. The four emission filters were alternated every 790 ms (with an exposure of 500 ms per frame), and the Ca^2+^ responses of individual cells lasted long enough (compared to the image acquisition rate) to be recorded in all the channels with approximately the same shape (Figure S3). The area under the Ca^2+^ response curves was integrated for each emission channel. When Aeq (emission peak at 465 nm, see Figure S2B) was expressed in HeLa cells and reconstituted with coelenterazine-h, 64% of the total counts (sum of the counts in the four channels) were recorded in the 481 nm channel, whereas 27%, 7% and 2% were in the 535, 595 and 640 nm channels, respectively (Table 2). In contrast, GA and CitA pairs displayed more than 70% of the recorded counts in the 535 nm channel, which overlapped both GFP and citrine emission peaks (Figure S2B). This result reflects a very efficient energy transfer from aequorin to GFP and citrine, as previously shown for GA [13]. Bioluminescence of mRA contributed 13% and 10% of the total counts to the 595 and 640 nm channels, respectively. However, 53% of the counts were still collected at 481 nm, indicating a moderate energy transfer from aequorin to mRFP1.2. The same conclusion was reached from previously reported mRA red/green ratio [18] or from the BRET spectrum of the related chimera RA [19]. A moderate energy transfer was also observed with tagRA, with 13% of the counts in the 595 nm band, corresponding to the TagRFP emission peak; however, it was no better than mRA. In turn, mOA showed the weakest energy transfer ability of all the tested BRET pairs, and was trivially different from Aeq. In sharp contrast with the latter, tdTA had as much as 28% and 12% of the counts in the 595 and 640 nm imaging channels, respectively. Furthermore, the distribution of the Ca^2+^-dependent tdTA counts in the four channels was similar in purified r-tdTA (Figure S1A) solutions *in vitro*: 39.8±4, 18.7±1.4, 29.3±4 and 12.3±2.5% of the total recorded counts in the 481, 535, 595 and 640 nm channels, respectively (mean±S.E., n = 3). Thus tdTA it is the aequorin fusion protein with most emission in the red band of the spectrum of the present series and of those reported before. Both the efficiency of BRET from donor to acceptor and the folding efficiency of tdTomato may contribute to tdTA enhanced red emission. Ca^2+^-induced bioluminescence reaction kinetics, intensity and spectrum strongly depend on the coelenterazine moiety and are, thus, affected by the analog used for reconstitution [9]. The observed spectral distribution of tdTA in HeLa cells (Table 2) did not change significantly when *native-* and *f-coelenterazine* were used for reconstitution instead of h-coelenterazine (data not shown) since they mainly affect aequorin Ca^2+^ affinity [9].

**Table 2:** Characterization of BRET efficiency and spectral contribution of BRET pairs.

	Spectroscopic properties of aequorin and various fluorescent proteins						Bioluminescence signal contribution (% of total) in each emission filter					
	λ_Abs_	λ_Em_	ε (M^−1^ cm^−1^)	QY	BR	BRET pairs	481 nm	535 nm	595 nm	640 nm	*J (*λ*)* (M^−1^ cm^−1^ nm^4^) 10^15^	*R_0_* (Å)
Aequorin	-	465	-	0.16	-	Aeq	64.1±2.4	27.1±2.0	6.5±1.4	2.4±0.6	-	-
GFP	488	509	56,000	0.60	34	GA	17.9±7.2	70.7±9.2	8.8±4.4	2.6±2.2	1.296	39.7
Citrine	516	529	77,000	0.76	59	CitA	10.8±3.0	72.2±5.0	13.8±2.7	3.2±1.3	1.052	38.3
mOrange	548	562	71,000	0.69	49	mOA	60.3±2.6	28.0±1.5	8.8±2.8	3.0±0.9	1.092	38.6
tdTomato	554	581	138,000	0.69	95	tdTA	41.5±2.4	18.9±1.9	27.5±2.2	12.1±2.5	2.654	44.7
TagRFP	555	584	100,000	0.48	48	tagRA	56.4±2.1	25.9±2.5	12.5±2.4	5.2±1.6	1.415	40.3
mRFP1.2	589	612	50,000	0.25	13	mRA	53.1±3.2	23.3±2.0	13.4±2.4	10.2±1.5	0.666	35.5

Absorbance (λ_Abs_) and emission maxima (λ_Em_), molar extinction coefficient (ε), emission quantum yield (QY), brightness (BR = ε
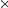
QY/1000), taken from references [22], [23], [31], [32], were used for the calculation of BRET parameters. The percentage contribution of aequorin (Aeq) bioluminescence and six BRET pairs into four emission channels was measured in HeLa cells expressing chimeras during Ca^2+^ responses (see *Materials and Methods*). Raw counts were corrected for filter bandwidth and transmittance, and also for camera sensitivity (manufacturer's data); the counts in each channel were divided by the sum of the counts in the four channels, and were expressed as a percentage. The average percentage of 15 to 49 cells ± S.D. is shown. Filters (Figure S2A) are named as their center wavelength. The theoretical BRET parameters, *J (*λ*)* and *R_0,_* are the spectral overlap integral and Förster distance (see *Materials and Methods* for details).

Table 2 also displays the calculated spectral overlap integral *J*(λ) and the Förster distance for a 50% energy transfer (*R_0_*) of the BRET chimeras, together with the literature values for peak absorbance, emission, extinction coefficient and quantum yield of the FPs. It is noteworthy that the more red-shifted the acceptor FP, the lower the *J*(λ) and *R_0_*, except for tagRA and tdTA. The latter had the highest *J*(λ) value and *R_0_* (44.7 Å) of them all. Two properties made tdTA exceptional: tdTomato's high molar extinction coefficient (it is a tandem dimer, a functional monomer but with twice the molecular weight) and its wide absorbance spectrum (Figure S4). Both the theoretical calculation of *R_0_* and the empirical four-point spectral analysis shown above suggest efficient BRET between aequorin and tdTomato. Therefore, tdTA was chosen for further characterization in Ca^2+^ imaging applications.

### Imaging of Physiological ATP-Dependent Ca^2+^ Mobilization in Single HeLa Cells

**Figure 2:**
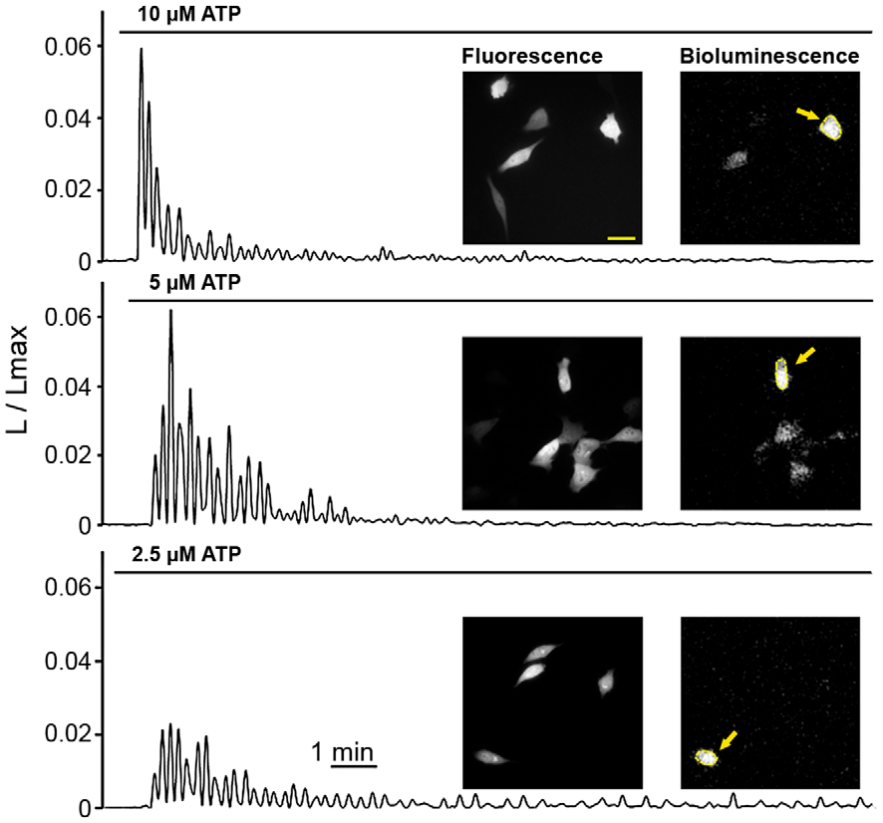
Imaging of ATP-induced Ca^2+^ oscillations in single live HeLa cells using tdTA. Fluorescence images of tdTA were taken before starting the time-lapse imaging of bioluminescence (0.25 frames/s). Cells were superfused with solutions containing ATP (as shown by bars) to induce cytosolic Ca^2+^ oscillations. The bioluminescence images presented were taken at the peak of Ca^2+^ responses, and regions of interest were defined on selected cells (arrows). Graphs show bioluminescence intensity (L) at each time point divided by the total remaining counts (Lmax), obtained by subsequent cell permeabilization (see *Materials and Methods*). L/Lmax is directly related to Ca^2+^ ion concentration. An EM-CCD camera was set for photon imaging mode 1. Bioluminescence images have 256×256 pixels, 0.76 µm/pixel. Scale bar equals 30 µm.

tdTA was used to image intracellular Ca^2+^ oscillations in HeLa cells in response to extracellular agonists. Receptor stimulation at physiologically relevant hormone concentrations leading to inositol-1,4,5-trisphosphate formation triggers regenerative Ca^2+^ release from the endoplasmic reticulum [33]. ATP is known to bind to P2Y receptors in HeLa cells, activate G-proteins and phospholipase C, leading to the accumulation of inositol-1,4,5-trisphosphate [34]. Extracellular superfusion of HeLa cells (Figure S1B) expressing tdTA with ATP produced cytoplasmic Ca^2+^ oscillations, which were observed in single cells (Figure 2 and Video S1). Lowering ATP concentration applied to cells from 10 µM to 2.5 µM resulted in smaller sized initial spikes, but less desensitization and a more sustained oscillatory response. Some individual cells expressing tdTA (as seen by fluorescence) did not display any Ca^2+^ activity (luminescence) upon ATP stimulation, but a strong signal was imaged upon their permeabilization with saponin at the end of the experiment, which brings millimolar external Ca^2+^ in contact with the probe. Control experiments with fluorescent indicator fluo-3 showed that not all the cells responded to the micromolar ATP concentration with Ca^2+^ oscillations (Figure S5), implying a different sensitivity of individual cells to ATP. Despite the lower quantum yield of aequorin-based measurements compared to fluorescence techniques, the shape of cells was well-distinguished by luminescence.

### Imaging of the Depolarization-Induced Ca^2+^ Response in Single Cultured Neurons

**Figure 3:**
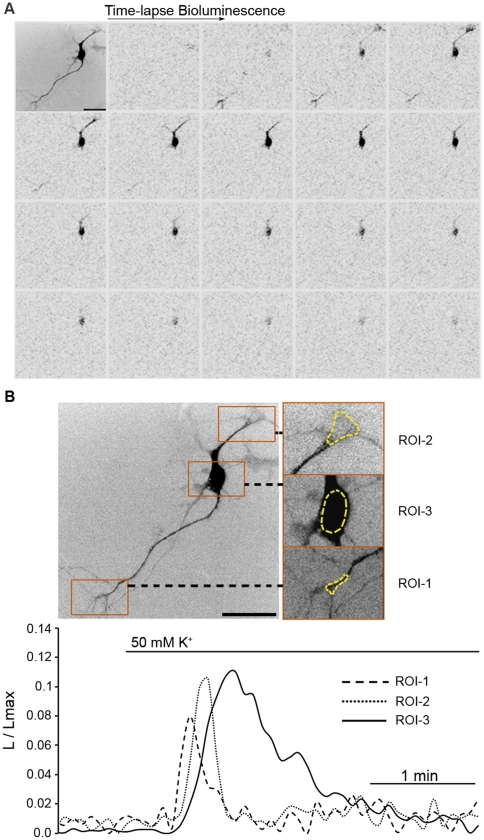
Bioluminescence imaging of the depolarization-induced Ca^2+^ response in a single mouse cortical neuron expressing tdTA. (**A**) A fluorescence image (top left) and a time-lapse series of bioluminescence images after superfusion with 50 mM K^+^ saline (0.2 frames/s) are shown. Black/white levels were inverted to improve contrast. (**B**) Graphical analysis of depolarization-induced Ca^2+^ response in three regions of interest (ROIs) on the cell body and neuronal projections marked by yellow traces on fluorescence images (3x zoom). ROI-1 and -2 correspond to distal neurites while ROI-3 encircles the soma. The graph shows the bioluminescence intensity expressed as L/Lmax over time (*Materials and Methods*). EM-CCD was set to photon-imaging mode 1. Bioluminescence images have 512×512 pixels, 0.38 µm/pixel. Scale bar equals 50 µm.

Mouse neurons in primary culture were used to investigate the ability of tdTA to report Ca^2+^ in cells with a more complex physiology and physical architecture than HeLa cells. A representative experiment of a cortical neuron expressing tdTA is shown in Figure 3. Perfusion with high K^+^ buffer produced membrane depolarization, the opening of voltage-dependent Ca^2+^ channels, Ca^2+^ entry and a bioluminescent tdTA response (Figure 3A). Several neurites were observed in the fluorescence and bioluminescence images; some projections were thin and barely visible by fluorescence, but bioluminescence was distinguishable over the background. By selecting regions of interest in different parts of the neuron (Figure 3B), we could monitor the local Ca^2+^ signal as a function of time. Time resolution of tdTA imaging (0.2 frames/s) was sufficient to show that in this neuron Ca^2+^-induced bioluminescence started at distal neurites and appeared later in the cell body. Another example of a Ca^2+^ response to depolarization is shown in a mouse mesencephalic neuron in culture (Video S2).

### Imaging Ca^2+^ in HeLa Cells Subcutaneously Inoculated into Mice

**Figure 4:**
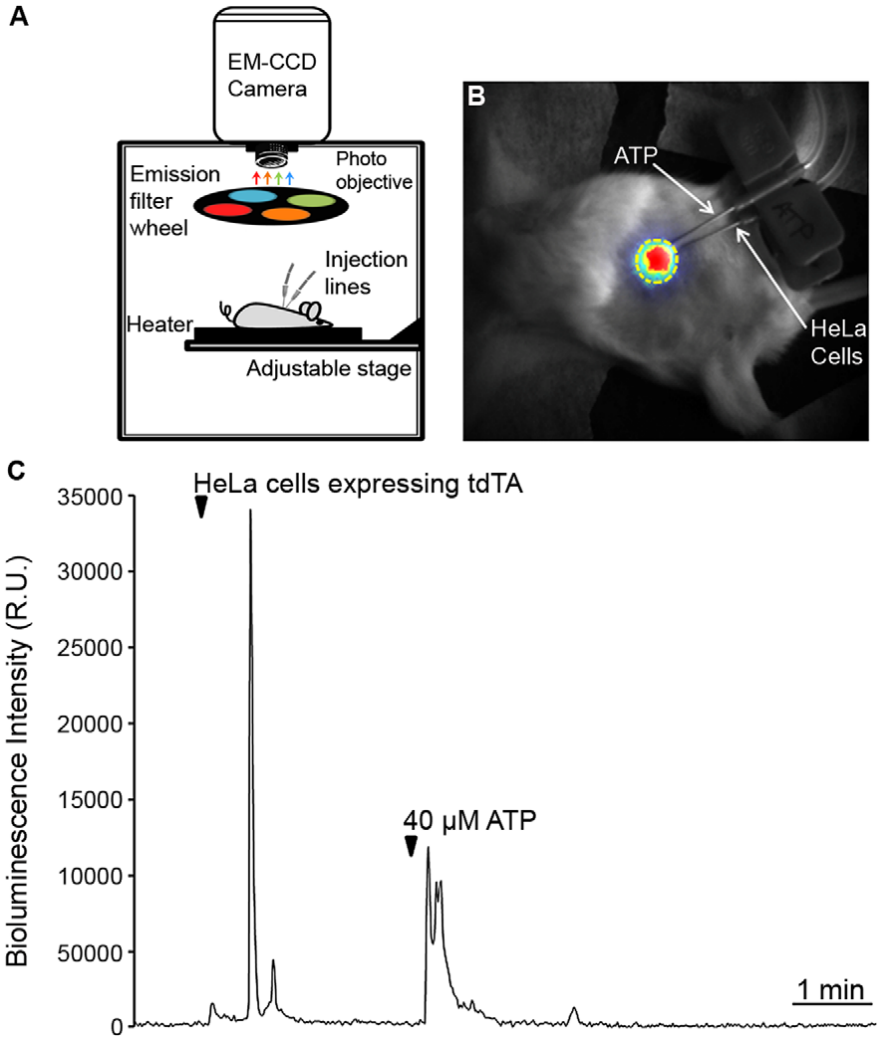
*In vivo* visualization of Ca^2+^ in HeLa cells subcutaneously inoculated into mice. (**A**) Bioluminescence setup for whole animal imaging. The setup consisted of a dark box containing a thermostated stage, an EM-CCD camera with a photo objective and an emission filterwheel. When needed, the filterwheel bearing four bandpass filters (481/34, 535/52, 595/40 and 640/50 nm) was placed between the mouse and the camera. Externally-controlled injection lines were prepared in the mouse before bioluminescence imaging. (**B**) Overlay of a reflection image (gray scale) of an anesthetized mouse (post-natal day 26) and the peak intensity of bioluminescence (RGB pseudo-color) after subcutaneous inoculation of tdTA-expressing HeLa cells. The region of interest used for the time-course analysis is marked in yellow. (**C**) Time-lapse graph of Ca^2+^-induced bioluminescence. Imaging started one minute before the inoculation of 70,000 HeLa cells (arrowhead). Three minutes later, 40 µM ATP was injected by a parallel needle. The EM-CCD camera was set to photon-imaging mode 1 and 1 frame/s.

A study of whole animal bioluminescence Ca^2+^ imaging in transgenic mice expressing GA has been reported recently [12]. Studies of the interaction of light with animal tissues have shown that light above 600 nm tends to cross tissues with minimum absorption and scattering, whereas blue/green light is more attenuated [21], [35]. In particular, oxyhemoglobin absorbance falls drastically at 600 nm, whereas infrared absorbance of water starts to rise at 900 nm [36]. Thus, a bioluminescent sensor for imaging Ca^2+^ in deep and vascularised tissues of animals should ideally have a strong emission in the imaging window between 600–900 nm. A large part of tdTA emission falls within this bandwidth (Table 2). HeLa cells expressing tdTA were isolated with a fluorescence-activated cell sorter and aequorin was reconstituted with h-coelenterazine. Those cells were deposited subcutaneously in mice to determine whether tdTA emission could be detected in superficial tissues. Two syringes connected by tubing to fine gauge needles were prepared, one containing the HeLa cell suspension and the other a buffered ATP solution. Needles were placed next to each other underneath the skin of an anesthetized mouse and image acquisition in a dark box setup was started (Figure 4A). Figure 4B–C shows the bioluminescence recorded upon injection of approximately 70,000 HeLa cells in a representative experiment. Inoculation of cells induced the mobilization of their internal Ca^2+^ and the registration of a large spike. The subsequent injection of extracellular ATP by the second syringe resulted in receptor stimulation and further light emission. Thus the Ca^2+^ activity of the tdTA-expressing cells injected into the SC tissue of live animals could be imaged with a temporal resolution of 1 frame/s.

### Visualization of r-tdTA Red Bioluminescence at Different Tissue Depth

**Figure 5:**
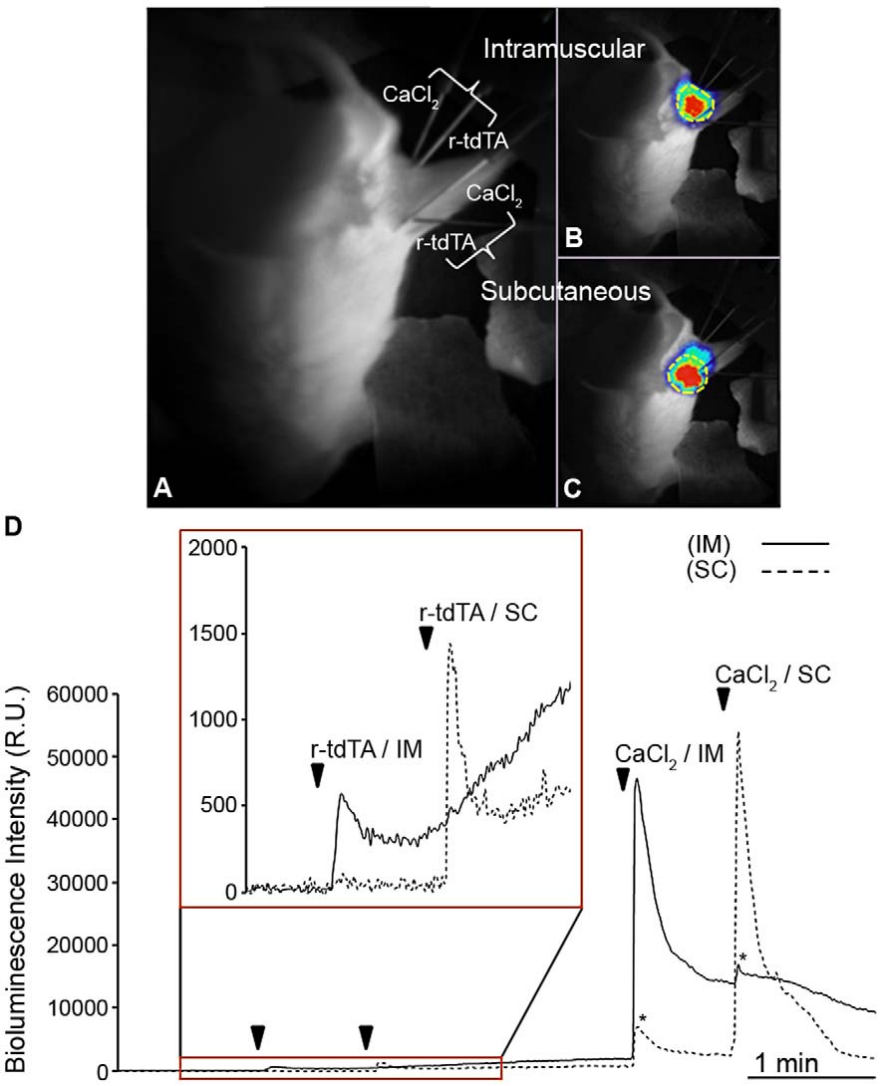
Ca^2+^-induced bioluminescence of recombinant tdTA injected into mouse subcutaneous and intramuscular tissues using the 595/40 nm emission filter. (**A**) Mouse dorsal view by white light reflection. One pair of injection tubes respectively containing r-tdTA (4 µg) and CaCl_2_ (15 mM) were placed in the intramuscular (IM) region and another pair in the subcutaneous (SC) region of an anesthetized mouse. (**B**) and (**C**) are image overlays of the reflection image (gray scale) and the peak of bioluminescence (RGB pseudo-color) upon injection of CaCl_2_ in the IM and SC regions, respectively. The regions of interest used for the time-lapse analysis are shown in yellow. (**D**) Time-course of Ca^2+^-induced bioluminescence in the IM and SC regions after injecting r-tdTA followed by CaCl_2_. The area in the rectangle with arrowheads is zoomed in the inset. Asterisks highlight the signal rise due to the spatial overlap between the IM and SC injection sites. The EM-CCD camera was set to photon-imaging mode 1 and the imaging rate of 1 frame/s.

Brûlet and coworkers compared the relative transmission of photoprotein-emitted light through tissues to find that RA light was better transmitted than GA light across all the mouse tissues examined [19]. For instance, Venus-aequorin and RA transmission were similar (and better than GA) in subcutaneous and subthoracic imaging under standardized conditions, but only RA (and not GA or Venus-aequorin) luminescence was detected across the skull. The probe tdTA will likely offer an even better tissue penetrating ability since we have shown that it emits a higher percentage of total light in the 595 and 640 nm channels than mRA. In the next set of experiments, recombinant purified r-tdTA, instead of cells expressing the probe, was injected into two different tissues, SC and intramuscular (IM). A pair of needles connected to syringes respectively containing reconstituted r-tdTA or CaCl_2_ were placed intramuscularly in the rear leg of an anesthetized mouse. Two other injection lines with the same amount of protein or CaCl_2_ were inserted in the SC tissue of the same animal about 1 cm apart from the IM needles (Figure 5A). Bioluminescence images were acquired using the 595/40 nm filter at 1 frame/s before and during the injection of r-tdTA into SC and IM tissue (Figure 5B–C). The graphical analysis of luminescence intensity at both injection sites showed that Ca^2+^-induced red emission could be detected just after the injection of r-tdTA at either site. This response had a rapid onset and proceeded for minutes, which is probably due to the diffusion of extracellular Ca^2+^ and the mixing with the protein solution in the imaging area (Figure 5D, inset). After injection of CaCl_2_ from the second set of syringes at the SC and IM sites, brighter responses were recorded (Figure 5D).

### Spectral Profile of tdTA Bioluminescence at Different Tissue Levels

**Figure 6:**
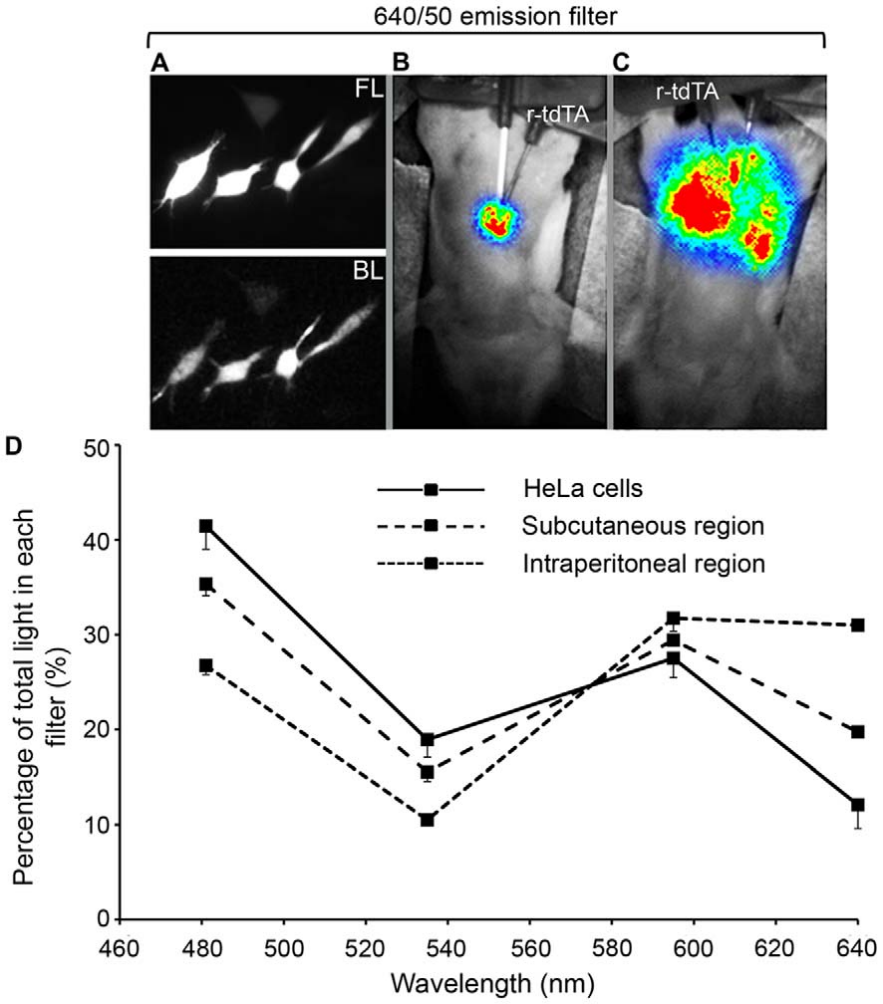
Percent contribution of tdTA bioluminescence into four emission channels at different tissue depth. (**A**) Fluorescence (FL) and Ca^2+^-induced bioluminescence (BL) of HeLa cells expressing tdTA. (**B**) and (**C**) are image overlays of the mouse reflection image (gray scale) and the Ca^2+^-induced bioluminescence (RGB pseudo-color) in the subcutaneous (B) and intraperitoneal (C) regions after injection of r-tdTA (4 µg). Ca^2+^-induced bioluminescence was imaged sequentially through four emission filters (481/34, 535/52, 595/40 and 640/50 nm) and was corrected for filter bandwidth and transmittance, as well as for camera sensitivity. (**D**) Relative contribution of bioluminescence into the emission channels (center wavelength). n = 2 in mice and n = 39 individual HeLa cells (±S.D.). EM-CCD was set to photon imaging mode 1 and 2 frames/s.

In the previous section, the signal from the r-tdTA injected into SC and IM tissues was strong enough to be recorded through a 595/40 nm filter, suggesting that a four-filter spectral profile could be obtained from superficial and deep tissues. Image acquisition was started before injecting reconstituted r-tdTA into anesthetized mice in SC or intraperitoneal (IP) regions (in different animals). After injecting r-tdTA, bioluminescence intensity was higher in IP (Figure 6C) compared to the SC region (Figure 6B), which is probably due to faster protein and Ca^2+^ diffusion in the IP space (Video S3). The percentage of light in the four channels shifted to the red with increasing tissue depth (Figure 6D). This was not likely to be caused by increased energy transfer efficiency, but should rather be attributed to a greater attenuation of blue/green light in the IP region since light has to cross the muscle and skin of the abdominal wall, whereas the SC injection is superficial [35]. This was in agreement with the observed contribution of luminescence of HeLa cells expressing tdTA into the same filters (Figure 6A and Table 2): the cell monolayer can be seen as a very thin “tissue” with minimum scattering and absorbance, showing a higher percentage of the total counts in the blue filter (Figure 6D).

## Discussion

In the present study, we have identified an efficient red FP acceptor for resonance energy transfer with the Ca^2+^-dependent photoprotein aequorin. We have obtained evidence that tdTA BRET chimera works well as a bioluminescent red Ca^2+^-sensor and that it has improved properties compared to previously described mRA [18] and RA [19].

In resonance energy transfer, the Förster distance (*R_0_*) of a pair of donor and acceptor chromophores is the distance at which the energy transfer efficiency is 50%. Large *R_0_* values are desirable because if donor-to-acceptor separation distance is greater than *R_0_*, transfer efficiency quickly decreases to zero. *R_0_* is given by the relative orientation of donor and acceptor transition dipoles (κ^2^), the quantum yield of the donor in the absence of the acceptor (*Q_D_*), and the degree of spectral overlap of donor emission and acceptor excitation *J*(λ). In turn, the spectral overlap integral depends on the extinction coefficient of the acceptor ε_A_(λ) [37]. In the fusions examined herein, we exchanged the acceptor; thus its extinction coefficient and spectral overlap with aequorin. Both the orientation and the effective distance between chromophores may have changed despite having used the same amino acids in the linker segment. Regarding the aim of moving aequorin luminescence into the red, two opposing forces are at work: long wavelength-emitting FPs are desirable, but usually their excitation spectra are also red shifted, resulting in less spectral overlap and less energy transfer. Consequently, an acceptor with a significantly large Stokes shift would be preferred. The calculated values of *R_0_* and the empirical four-point spectral analysis in living cells demonstrated that tdTA had the highest BRET efficiency and provided the largest number of counts in the 595 and 640 nm channels of all the fusions examined. In a recent study on FRET from a donor to multiple acceptor FPs, it has been shown that the amount of energy transfer observed was larger than the efficiency predicted from the sum of transfer rates to each acceptor molecule [38]. Since tdTomato is a tandem dimer, it could behave in a similar way as a whole system rather than as individual monomers. Therefore, the broad absorbance spectrum of tdTomato and its dimeric nature, responsible for its high molar extinction coefficient (138,000 M^−1^ cm^−1^), contribute to the large calculated spectral overlap *J*(λ) and Förster distance *R_0_* of tdTA. In addition to the factors contributing to BRET, the high quantum yield of tdTomato and its relatively large Stokes shift (27 nm) further improved the overall brightness of tdTA in the red emission band. Finally, tdTA took the shortest time to form its red fluorochrome of all the chimeras (Figure 1B) in agreement with previously reported FP maturation data [6], [23].

When comparing the *J*(λ) and *R_0_* parameters calculated herein (Table 2) with those of some FRET pairs [39], BRET gives generally lower *R_0_* values, which is most likely due to aequorin's low quantum yield. In our probes, the lowest *R_0_* was observed for mRA (35.5 Å) caused by the relatively low extinction coefficient of mRFP1.2 and its small spectral overlap with aequorin (mRFP1.2 is the most red-shifted acceptor). The *J*(λ) and *R_0_* of the remaining BRET constructs were more similar.

The chimeras GA and CitA showed a very efficient energy transfer from the donor aequorin (Table 2) in agreement with previous reports on GA [13], [17] and yellow-emitting Venus-aequorin [19]. Interestingly, they displayed more energy transfer than tdTA in spite of having lower *R_0_* values (GA 39.7 Å, CitA 38.3 Å) than tdTA (44.7 Å). A Ca^2+^-induced interaction has been reported between donor and acceptor modules in a *wild-type GFP*-aequorin fusion (with a 19 amino-acid flexible linker), which also resulted in a red shift of wild-type GFP absorbance (increased 475 nm and decreased 398 nm bands), thus a better overlapping with aequorin [40]. We suggest that *Aequorea*-derived FPs, such as GFP, citrine and Venus, may share this weak Ca^2+^-dependent interaction with aequorin, which would lead to a shorter distance and/or a more favorable orientation in the transfer complex; FPs derived from other organisms (also altered in many residues by mutagenesis) may lack this ‘privileged’ relation.

The four-emission filter approach employed herein was able to reproduce the emission profile of well-characterized GFP fluorescence and aequorin luminescence. It did not provide fine spectral details of the BRET pairs studied [13], [19], but allowed us to estimate the combined effect of BRET efficiency, peak emission wavelength and intrinsic brightness of the acceptor, plus overall maturation of the chimera, directly in the biological system under study. For instance, comparing the percentage of light contribution of GA and CitA into the 481 and 595 nm channels (Table 2) sufficed to distinguish the dissimilar luminescence spectra caused by the 20 nm emission red shift of citrine compared to GFP. In amplitude and frequency terms, very different Ca^2+^ responses were observed using tdTA in cellular systems. The Ca^2+^ oscillations produced by physiological stimulation of the HeLa cells with ATP were well detected by tdTA, whereas application of high [K^+^] to neurons caused immediate Ca^2+^-induced bioluminescence, which could be well resolved in time (0.2 frames/s) and space (0.38 µm per pixel) in small cell projections.

The issue of light propagation through tissues is not only a concern for bioluminescence imaging of Ca^2+^ in intact organisms, but also for fluorescence imaging [20]. The latter is aggravated by the fact that light must reach the target probe at a sufficiently high intensity to allow excitation; few FPs that can be excited between 600 and 900 nm have been produced [41]. Although we did not record physiological responses with tdTA in mice, we were able to detect Ca^2+^ responses by the stimulation of membrane receptors in cells expressing tdTA deposited subcutaneously. Given its significant contribution of counts in the red channels compared to other BRET pairs, tdTA seems a promising probe for imaging Ca^2+^ activity of specific groups of cells in organisms expressing the transgene.

## Materials and Methods

### Hybrid Gene Construction

Two systems, pTriEx-4 (Novagen) and pcDNA3 (Invitrogen), were used to express the hybrid genes in bacterial or mammalian cells, respectively. The starting GA cDNA, used in ref. [18], was developed by Dr. P. Brûlet [13]. The GFP in GA was replaced with five different FP acceptors. Citrine, mOrange, mRFP1.2 and tdTomato [23], [31] were a gift from Dr. R.Y. Tsien, while TagRFP [22] was provided by Dr. T.W.J. Gadella. FP cDNA sequences were PCR-amplified using oligonucleotides that allowed the insertion of a 5′ HindIII restriction site and a Kozak sequence for optimized mammalian translation (5′-GACAGTAAGCTTGCCACCATGGTGAGCAAGGGC-3′) and the removal of the stop codon and insertion of a 3′ KpnI site (5′-GATCATGGTACCCTTGTACAGCTCGTC-3′). The PCR fragment containing the FP gene was digested with HindIII/KpnI and subcloned instead of GFP in the digested GA pcDNA3 vector. The same flexible 17-amino acid linker (GTELYKSGGSGSGGQSG, in single-letter notation) was conserved in all the BRET constructs, and the aequorin C-terminus was kept intact [25]. Three recombinant proteins, r-Aeq, r-tdTA and r-CitA, were chosen for bacterial expression and purification, and were cloned as follows: tdTA and CitA in pcDNA3 were excised by digestion (HindIII/XhoI) and subcloned into the pTriEx-4 vector. To construct expression vectors coding for aequorin alone, a Kpn2I site was created by mutating the HindIII site upstream of tdTA in pcDNA3 and pTriEx-4 using the multi-site directed mutagenesis kit (Stratagene) with oligonucleotide 5′-GACAGTAAGCTTGCCACCATGGTCCGGAAGGGC-3′. Subsequently, the tdTomato-linker cDNA fragment was removed by digestion with Kpn2I; religation gave rise to pcDNA3/pTriEx-4 vectors bearing aequorin alone. The N-terminal His-tag motif in pTriEx-4 allowed protein purification. Restriction enzymes were obtained from Fermentas. *E. coli* strains DH5α (Invitrogen) were used for plasmid amplification and the Midi-prep system kit (Promega) was employed for plasmid extraction and purification. All the sequences were verified by DNA sequencing (Macrogen).

### Cell Culture and Transfection

HeLa cells were cultured in Dulbecco's modified Eagle's medium (Lonza) supplemented with 10% fetal bovine serum (FBS), 2 mM L-glutamine (Lonza) and 100 U/ml penicillin/streptomycin (Lonza) at 37°C with 5% CO_2_. Primary cultures of cortical and mesencephalic neurons were obtained from fetal (17-days gestation) Swiss albino white mice (species: balb/c). Pieces of cerebral tissue were incubated with 0.25% trypsin for 20–25 min at 37°C in Hank's Balanced Salt Solution (HBSS; Lonza) and triturated into single-cell suspension in HBSS supplemented with 10% FBS and 0.1 mg/ml DNAse. Serum was removed by washing three times with HBSS. The single-cell suspension was plated on glass coverslips coated with poli L-lysine at a density of 2×10^5^ cells/cm^2^. The culture medium was Neurobasal (Gibco) supplemented by 10% FBS, 2% B27 supplement (Gibco), 2 mM glutamine and 1 mg/mL gentamycin. Cultures were maintained at 37°C with 5% CO_2_ and a humidified atmosphere. One day later, cells were shifted to a maintenance medium similar to the plating media, but lacking FBS. Two days after plating, non-neuronal cell division was halted by 1–3 days exposure to 1 µM cytosine arabinoside. Neurons were identified by their morphology, which was very different from that of astrocytes cultured with an appropriate protocol. HeLa and neuronal cells were transfected 2 and 6 days after cell seeding, respectively, with Lipofectamine 2000 (Invitrogen) according to the manufacturer's protocol.

### Hybrid Protein Expression and Purification from *E. coli*

For the heterologous expression of the His-tagged recombinant r-Aeq, r-tdTA and r-CitA, the *E. coli* strain BL21(DE3)pLysS (Novagen) cells bearing the target plasmid were grown to saturation in 4 ml of LB medium with ampicillin (100 µg/ml) at 37°C, and cultures were diluted into 50 ml of fresh medium. Cultures were grown until cell density reached an absorbance of 0.4 at 600 nm, diluted into 500 ml of fresh medium and grown until absorbance reached 0.6. The expression of the hybrid proteins was induced by the addition of 1 mM isopropyl-β-D-thiogalactopyranoside. After incubation for 4 h at 30°C, cells were harvested by centrifugation at 5,000× g for 5 min at 4°C. All the purification steps were performed at 4°C or on ice. Cells were washed once in 20 mM Tris-HCl buffer (Ph 7.5) and suspended in 30 ml lysis buffer (50 mM phosphate buffer, 300 mM NaCl, 10 mM imidazol, 0.1% Triton X-100, 20 mM β-mercaptoethanol, 1 mM phenylmethylsulfonyl fluoride, 1 protease-inhibitor cocktail tablet (complete-Mini, EDTA-Free, Roche), pH 7.5). Cells were disrupted by sonication (Ultraschallprozessor, UP 200 S) with 15 rounds of 10 pulses each, allowing for a rest of 20–30 s between rounds and using 0.5 cycles, 50 A. Unbroken cells and cell debris were removed by centrifugation at 16,000× g for 30 min at 4°C. Subsequent protein purification steps were in accordance with the manufacturer's protocol (Ni-NTA HisBind Resins; Novagen). Eluted hybrid protein was quantified using the BCA protein assay (Thermo Scientific) with absorbance at 280 nm (Nanodrop).

### *In vitro* Reconstitution of Apoaequorin

Freshly eluted apoaequorin solution was reconstituted by adding 5 µM coelenterazine-h (Prolume or NanolightTech) or coelenterazine-f (Invitrogen), 10 mM dithiothreitol and 5 mM ethylene glycol tetraacetic acid, followed by a 1-hour incubation at 4°C using moderate orbital shaking [42]. The protein preparation was concentrated by gel filtration through a column of centrifugal filters (Microcon YM-30, Millipore) and was buffer-changed (150 mM Tris-HCl, 100 mM NaCl and 1 mM ethylene glycol tetraacetic acid, pH 8).

### Microscopy Imaging

For image acquisition at 22–24°C, coverslips with HeLa cells or neurons were transferred to an Attofluor microscopy chamber (Molecular Probes), and the medium was replaced with HBSS. A saline superfusion system was used to exchange solutions during imaging; input was driven by gravity and output was obtained by suction from a fish tank pump (inverted flow). An epifluorescence inverted microscope (DMIRE-2, Leica) with a PlanApo 40x N.A. 1.25 oil immersion objective was used for both the fluorescence and bioluminescence imaging of cells. The detector was an electron-multiplying charge-coupled device (EM-CCD) camera (EMC9100-13, Hamamatsu Photonics) cooled to −65°C; a proprietary ultrasensitive mode, called the ‘photon imaging mode’, was used for bioluminescence imaging. In contrast with fluorescence, imaging emitted light was collected continuously for bioluminescence, except for the time taken for the image transfer from the camera to the computer. The CCD camera was controlled and images were acquired using the AquaCosmos 2.6 software (Hamamatsu Photonics). Images were corrected for background, and regions of interest were selected manually.

#### Fluorescence Imaging and Characterization of Red FP Maturation

A polychromatic light source (C7773, Hamamatsu) was used for excitation. Fluorescence images were taken using two defined channels. The green channel, used for GFP and citrine, was obtained with a polychromator set at 500 nm and a filtercube BrightLine HC-YFP (Semrock) (HC500/24 nm exciter, 520 nm beamsplitter and a HC542/27 nm emitter, center wavelength/bandwidth). The red channel, used for mOrange, tdTomato, TagRFP and mRFP1.2, used a polychromator set at 550 nm, a 570DRLP beamsplitter, and a 595/40 nm emission filter placed in the filterwheel under the microscope (filters were obtained from Omega Optical). Peripheral control of the polychromator and the emission filterwheel was performed with the AquaCosmos software. The contribution of a green component during the maturation of red FPs (mOrange, tdTomato, TagRFP and mRFP1.2) in fusion proteins mOA, tdTA, TagRA and mRA was estimated by comparing the green to red emission ratios in the single HeLa cells expressing chimeras at 8, 15, 24, 30, 45, 52 and 72 hours post-transfection. Imaging was performed with the above-defined green and red channels.

#### Single Cell Bioluminescence Imaging of Ca^2+^

Before each experiment, HeLa cells or neurons were rinsed with phosphate-buffered saline, and apoaequorin was reconstituted by incubating cells with 5 µM coelenterazine-h (Invitrogen) in OptiMEM I medium (Gibco) supplemented with 1% FBS for 2 hours at 37°C and 5% CO_2_ [4]. The imaging field was selected by fluorescence (or by transmitted light should Aeq be expressed alone). Bioluminescence imaging of single cells was carried out in the dark to reduce the background by using a lightproof cover over the microscope. The Ca^2+^-induced bioluminescence signal collected by the objective went through an emission filter held in a filterwheel (or through an empty position with no filter, where indicated), and was captured by the EM- CCD camera (setup is shown in Figure S1B).

In the experiments with HeLa cells and neurons, bioluminescence counts were converted and displayed as *L/Lmax*, a mathematical term previously developed that is directly correlated to Ca^2+^ ion concentration [43], where *L* is the measured bioluminescence intensity at each time and *Lmax* is the remaining total amount of counts from that time to the end of the experiment. To obtain total light from the remaining active aequorin, cells were permeabilized with 0.01% saponin at the end of each experiment (Quillaja Bark, Sigma) to allow Ca^2+^ entry from the extracellular medium (1.3 mM Ca^2+^). We observed that it is important to avoid fast Ca^2+^ entry upon cell permeabilization in order to prevent rapid bursts of photons which would saturate the detector and compromise the calculation of *Lmax*. Various reagent concentrations were tested to slow down the consumption rate of the remaining aequorin.

### Characterization of the Emission Profile of FP-Aequorin Fusions by the Four-Channel Approach

The HeLa cells transiently expressing the different BRET pairs and reconstituted with coelenterazine-h (Prolume or NanolightTech) were superfused with 0.01% saponin in HBSS with 1.3 mM CaCl_2_. The Ca^2+^-induced bioluminescence signal was imaged sequentially through four emission filters held in the filterwheel: 481/34, 535/52, 595/40 and 640/50 nm (center wavelength/bandwidth, Figure S2A). Filters (Omega Optical except 595/40, which was obtained from Chroma) were selected for spectral separation and for covering a wide range of the visible spectrum (464–665 nm). The contribution of bioluminescence into these four channels was quantified in each single cell, and counts were corrected for background and normalized for filter bandwidth, transmittance and for the spectral sensitivity of the EM-CCD camera (according to the manufactureŕs data). An example of the graphical data analysis is shown in Figure S3.

To validate this quantification method, we reconstructed the well-known fluorescence emission spectrum of GFP and the bioluminescence spectrum of Aeq by means of the above four channel measurements and corrections (Table 1). The predicted signal contribution into each channel was calculated from the integral of filter transmission and the known published emission spectra of GFP and Aeq (Figure S2). The experimental data for Aeq expressed in HeLa cells were obtained as for the BRET pairs described above by integrating the bioluminescence contribution into the four channels during a Ca^2+^ response (as in Figure S3). GFP fluorescence emission was recorded with excitation at 420 nm in the transfected HeLa cells. Raw counts were corrected for filter bandwidth/transmittance and camera sensitivity; the counts in each channel were divided by the sum of the counts in the four channels and were expressed as a percentage.

### Calculation of BRET Critical Distance *R_0_*

The Förster radius (*R_0_*), at which half the energy is transferred by BRET, was calculated according to Equations 1 and 2 [37]:
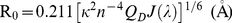
(1)

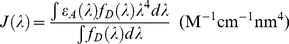
(2)


where *J*(λ) is the spectral overlap integral, *Q_D_* is the donor quantum yield, *n* is the refractive index of the medium (assumed to be 1.33 of water), *κ^2^* is the orientation factor (assumed to be 2/3 corresponding to the random donor and acceptor orientation), ε*_A_*(λ) is the (wavelength-dependent) molar extinction coefficient of the acceptor (in M^−1^cm^−1^), *f_D_*(λ) is the (wavelength-dependent) donor emission intensity (in arbitrary units) and λ is the wavelength (in nm).

### Fluorescence Activated Cell Sorting

The HeLa cells expressing tdTA were isolated using a flow cytometer and a cell sorter (Cytopeia INFLUX), and were used for mice experiments. Thirty hours after transfection, HeLa cells were washed twice with phosphate-buffered saline solution, detached from the plate using 200 mg/l tripsin EDTA (BioWhittaker) and resuspended in 5 ml Dulbecco's modified Eagle's medium without phenol red (Lonza). Sorting was carried out using a 542 nm laser excitation and a 600/50 nm emission filter; sorted cells were incubated with h-coelenterazine to reconstitute aequorin.

### Preparing Mice for *in vivo* Ca^2+^ Imaging

CD-1 IGS mice (Charles River) were maintained in the animal facility at the Albacete Medical School. Animal's ages varied from P14 to P45. Mice were anesthetized by an intraperitoneal injection of a freshly prepared solution of 75 mg/kg ketamine (Pfizer) and 10 mg/kg xylazine (Calier). All efforts were made to minimize animal suffering. An area of hair was removed to facilitate imaging and mice were kept warm during the experimental study. Whole animal Ca^2+^ bioluminescence imaging was performed in a dark box using the same camera and filterwheel as described for live cell imaging (setup shown in Figure 4A). Injection needles were preloaded with adequate solutions and collocated in subcutaneous, intraperitoneal or intramuscular regions before starting image acquisition. Injection of HeLa cells, purified r-tdTA, ATP, or CaCl_2_ solutions was performed as indicated. Experimental procedures were in agreement with the European Union Guidelines for the Care and Use of Laboratory Animals (Council Directive 86/609/EEC). The protocol was approved by the Committee on the Ethics of Animal Experiments of the University of Castilla-La Mancha (January 10th, 2008).

## Supporting Information

Figure S1 **Recombinant r-tdTA construction and microscopy bioluminescence imaging setup.** (**A**) Scheme of the tdTA construct for bacterial expression and purification using the His-tag motif in vector pTriEx-4. (**B**) Coverslips with the cells expressing FP-aequorin fusions were superfused with solutions in a chamber on the stage of an inverted microscope. A filterwheel containing four bandpass filters (481/34, 535/52, 595/40 and 640/50 nm) was placed between the objective and the EM-CCD camera.
(TIF)

Figure S2 **Emission filters and emission spectra of aequorin and various FPs.** (**A**) Transmittance profile of the bandpass interference filters and EM-CCD sensitivity used for characterizing FP-aequorin fusions in HeLa cells and mice. Filters are defined as the center wavelength and bandwidth at half-maximal transmittance. (**B**) Normalized emission spectra of h-aequorin and various acceptor FPs. The h-Aequorin, GFP, mRFP1.2 and TagRFP data were taken from references [22], [23], [31], [32].
(TIF)

Figure S3 **Graphical data analysis of bioluminescence spectral contribution of tdTA and CitA in HeLa cells using the four-channel imaging approach.** (**A**) tdTA (**B**) CitA. The Ca^2+^ response of the HeLa cells expressing either chimera during cell permeabilization with saponin was imaged in the four channels in sequential order: a 481-nm channel image was followed 0.8 s later by a 535-nm image, then 595-nm and finally 640-nm images. This cycle was repeated continuously during the experiment. The shape of the Ca^2+^ response was similar in the four channels. The area under the Ca^2+^ response curves (gray shading) was integrated for each filter.
(TIF)

Figure S4 **Spectral overlap between h-aequorin bioluminescence and the absorbance spectra of various acceptor FPs.** The h-aequorin, GFP, mRFP1.2 and TagRFP data were taken from references [22], [23], [31], [32].
(TIF)

Figure S5 **Fluorescence imaging of ATP-induced Ca^2+^ oscillations in live HeLa cells using Fluo-3.** HeLa cells were incubated with 1 µM Fluo3-AM (Molecular Probes) for 45 minutes at room temperature and washed. Image acquisition (500 nm excitation, 122 ms exposure and 2-second image interval using a 40x objective/1.25 NA) started before applying 10 µM ATP in HBSS solution (horizontal bar). ROIs 1 and 2 represent examples of two cells with different Ca^2+^ responses. Image: 512×512 pixels. Scale bar equals 40 µm.
(TIF)

Video S1 **Ca^2+^ oscillations in HeLa cells induced by the external application of 5** *µ* **M ATP measured with tdTA.** Fluorescence and bioluminescence are shown. Time units are minutes:seconds.
(AVI)

Video S2 **tdTA reports Ca^2+^ activity in a mesencephalic neuron upon depolarization with 50 mM K^+^.**
(AVI)

Video S3 **Bioluminescence response to local Ca^2+^ after intraperitoneal injection of recombinant tdTA in an anesthetized mouse.** An overlay of the mouse reflection image (gray scale) and Ca^2+^-induced bioluminescence (red pseudocolor) is shown.
(AVI)

## References

[1] Feske S, Giltnane J, Dolmetsch R, Staudt LM, Rao A (2001). Gene regulation mediated by calcium signals in T lymphocytes.. Nat Immunol.

[2] Ghosh A, Greenberg ME (1995). Calcium signaling in neurons: molecular mechanisms and cellular consequences.. Science.

[3] Ruegg JC (1987). Calcium regulation of muscle contraction: the molecular regulation mechanisms of contracility.. Naturwissenschaften.

[4] Creton R, Kreiling JA, Jaffe LF (1999). Calcium imaging with chemiluminescence.. Microsc Res Tech.

[5] Grynkiewicz G, Poenie M, Tsien RY (1985). A New Generation of Ca^2+^ Indicators with Greatly Improved Fluorescence Properties.. JBC.

[6] Shaner NC, Steinbach PA, Tsien RY (2005). A guide to choosing fluorescent proteins.. NatMethods.

[7] Miyawaki A, Llopis J, Heim R, McCaffery JM, Adams JA (1997). Fluorescent indicators for Ca^2+^ based on green fluorescent proteins and calmodulin.. Nature.

[8] Ohmiya Y, Hirano T (1996). Shining the light: the mechanism of the bioluminescence reaction of calcium-binding photoproteins.. ChemBiol.

[9] Shimomura O (1995). Cause of spectral variation in the luminescence of semisynthetic aequorins.. BiochemJ 306 (Pt.

[10] Montero M, Brini M, Marsault R, Alvarez J, Sitia R (1995). Monitoring dynamic changes in free Ca^2+^ concentration in the endoplasmic reticulum of intact cells.. EMBO J.

[11] Alonso MT, Manjarres IM, Garcia-Sancho J (2009). Modulation of calcium signalling by intracellular organelles seen with targeted aequorins.. Acta Physiol (Oxf).

[12] Rogers KL, Picaud S, Roncali E, Boisgard R, Colasante C (2007). Non-invasive in vivo imaging of calcium signaling in mice.. PLoS One.

[13] Baubet V, Le MH, Campbell AK, Lucas-Meunier E, Fossier P (2000). Chimeric green fluorescent protein-aequorin as bioluminescent Ca^2+^ reporters at the single-cell level.. ProcNatlAcadSciUSA.

[14] Shimomura O, Kishi Y, Inouye S (1993). The relative rate of aequorin regeneration from apoaequorin and coelenterazine analogues.. Biochem J 296 (Pt.

[15] Shimomura O, Johnson FH, Saiga Y (1962). Extraction, purification and properties of aequorin, a bioluminescent protein from the luminous hydromedusan, Aequorea.. J Cell Comp Physiol.

[16] Morise H, Shimomura O, Johnson FH, Winant J (1974). Intermolecular energy transfer in the bioluminescent system of Aequorea.. Biochemistry.

[17] Waud JP, Bermudez Fajardo A, Sudhaharan T, Trimby AR, Jeffery J (2001). Measurement of proteases using chemiluminescence-resonance-energy-transfer chimaeras between green fluorescent protein and aequorin.. Biochem J.

[18] Manjarres IM, Chamero P, Domingo B, Molina F, Llopis J (2008). Red and green aequorins for simultaneous monitoring of Ca^2+^ signals from two different organelles.. Pflugers Arch.

[19] Curie T, Rogers KL, Colasante C, Brulet P (2007). Red-shifted aequorin-based bioluminescent reporters for in vivo imaging of Ca^2+^ signaling.. Mol Imaging.

[20] Weissleder R, Ntziachristos V (2003). Shedding light onto live molecular targets.. NatMed.

[21] Zhao H, Doyle TC, Coquoz O, Kalish F, Rice BW (2005). Emission spectra of bioluminescent reporters and interaction with mammalian tissue determine the sensitivity of detection in vivo.. JBiomedOpt.

[22] Merzlyak EM, Goedhart J, Shcherbo D, Bulina ME, Shcheglov AS (2007). Bright monomeric red fluorescent protein with an extended fluorescence lifetime.. NatMethods.

[23] Shaner NC, Campbell RE, Steinbach PA, Giepmans BN, Palmer AE (2004). Improved monomeric red, orange and yellow fluorescent proteins derived from Discosoma sp. red fluorescent protein.. NatBiotechnol.

[24] Griesbeck O, Baird GS, Campbell RE, Zacharias DA, Tsien RY (2001). Reducing the environmental sensitivity of yellow fluorescent protein. Mechanism and applications.. J Biol Chem.

[25] Nomura M, Inouye S, Ohmiya Y, Tsuji FI (1991). A C-terminal proline is required for bioluminescence of the Ca(^2+^)-binding photoprotein, aequorin.. FEBS Lett.

[26] Badminton MN, Sala-Newby GB, Kendall JM, Campbell AK (1995). Differences in stability of recombinant apoaequorin within subcellular compartments.. BiochemBiophysResCommun.

[27] Gross LA, Baird GS, Hoffman RC, Baldridge KK, Tsien RY (2000). The structure of the chromophore within DsRed, a red fluorescent protein from coral.. Proc Natl Acad Sci U S A.

[28] Strack RL, Strongin DE, Mets L, Glick BS, Keenan RJ (2010). Chromophore formation in DsRed occurs by a branched pathway.. J Am Chem Soc.

[29] Subach OM, Malashkevich VN, Zencheck WD, Morozova KS, Piatkevich KD (2010). Structural characterization of acylimine-containing blue and red chromophores in mTagBFP and TagRFP fluorescent proteins.. Chem Biol.

[30] Wachter RM, Watkins JL, Kim H (2010). Mechanistic diversity of red fluorescence acquisition by GFP-like proteins.. Biochemistry.

[31] Campbell RE, Tour O, Palmer AE, Steinbach PA, Baird GS (2002). A monomeric red fluorescent protein.. ProcNatlAcadSciUSA.

[32] Inouye S, Sasaki S (2006). Blue fluorescent protein from the calcium-sensitive photoprotein aequorin: catalytic properties for the oxidation of coelenterazine as an oxygenase.. FEBS Lett.

[33] Tsien RW, Tsien RY (1990). Calcium channels, stores, and oscillations.. AnnuRevCell Biol.

[34] Okuda A, Furuya K, Kiyohara T (2003). ATP-induced calcium oscillations and change of P2Y subtypes with culture conditions in HeLa cells.. Cell BiochemFunct.

[35] Caysa H, Jacob R, Muther N, Branchini B, Messerle M (2009). A redshifted codon-optimized firefly luciferase is a sensitive reporter for bioluminescence imaging.. Photochem Photobiol Sci.

[36] Stamatas GN, Southall M, Kollias N (2006). In vivo monitoring of cutaneous edema using spectral imaging in the visible and near infrared.. J Invest Dermatol.

[37] Lakowicz JR (2006). Principles of Fluorescence Spectroscopy..

[38] Koushik SV, Blank PS, Vogel SS (2009). Anomalous surplus energy transfer observed with multiple FRET acceptors.. PLoS One.

[39] Hink MA, Visser NV, Borst JW, Hoek Av, Visser AJWG (2004). Practical use of corrected fluorescence excitation and emission spectra of fluorescent proteins in Förster Resonance Energy Transfer (FRET) studies.. J FLUORESC.

[40] Gorokhovatsky AY, Marchenkov VV, Rudenko NV, Ivashina TV, Ksenzenko VN (2004). Fusion of Aequorea victoria GFP and aequorin provides their Ca(^2+^)-induced interaction that results in red shift of GFP absorption and efficient bioluminescence energy transfer.. Biochem Biophys Res Commun.

[41] Lin MZ, McKeown MR, Ng HL, Aguilera TA, Shaner NC (2009). Autofluorescent proteins with excitation in the optical window for intravital imaging in mammals.. ChemBiol.

[42] Prasher D, McCann RO, Cormier MJ (1985). Cloning and expression of the cDNA coding for aequorin, a bioluminescent calcium-binding protein.. BiochemBiophysResCommun.

[43] Allen DG, Blinks JR, Prendergast FG (1977). Aequorin luminescence: relation of light emission to calcium concentration--a calcium-independent component.. Science.

