# Supplementary figures and images for: Red Fluorescent Protein-Aequorin Fusions as Improved Bioluminescent Ca2+ Reporters in Single Cells and Mice
Source: PLoS One. 2011 May 11;6(5):e19520. doi: 10.1371/journal.pone.0019520 (PMC3092744; doi:10.1371/journal.pone.0019520)

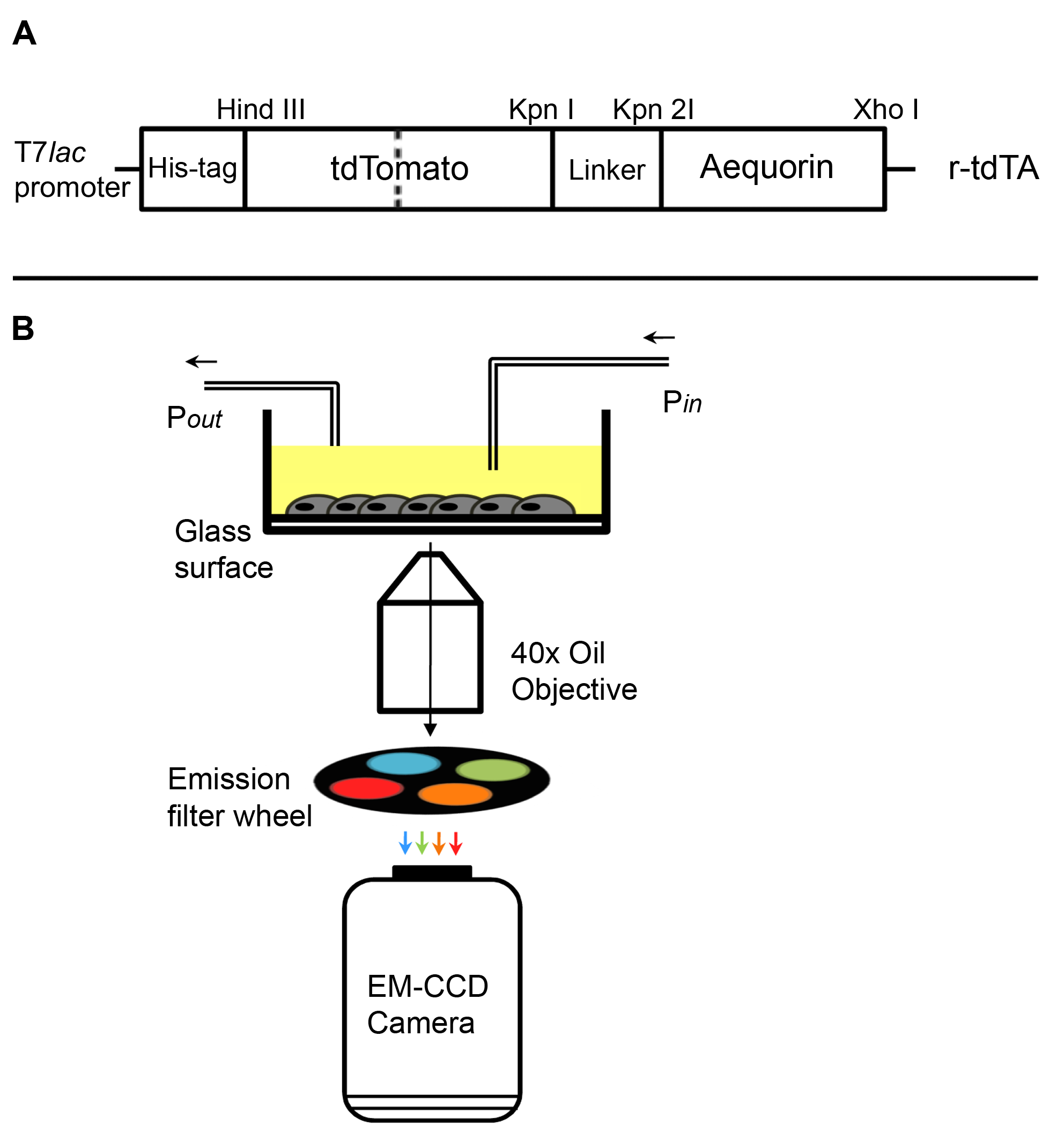

Supplement: Figure S1 — Recombinant r-tdTA construction and microscopy bioluminescence imaging setup. (A) Scheme of the tdTA construct for bacterial expression and purification using the His-tag motif in vector pTriEx-4. (B) Coverslips with the cells expressing FP-aequorin fusions were superfused with solutions in a chamber on the stage of an inverted microscope. A filterwheel containing four bandpass filters (481/34, 535/52, 595/40 and 640/50 nm) was placed between the objective and the EM-CCD camera. (TIF) [file pone.0019520.s001.tif]

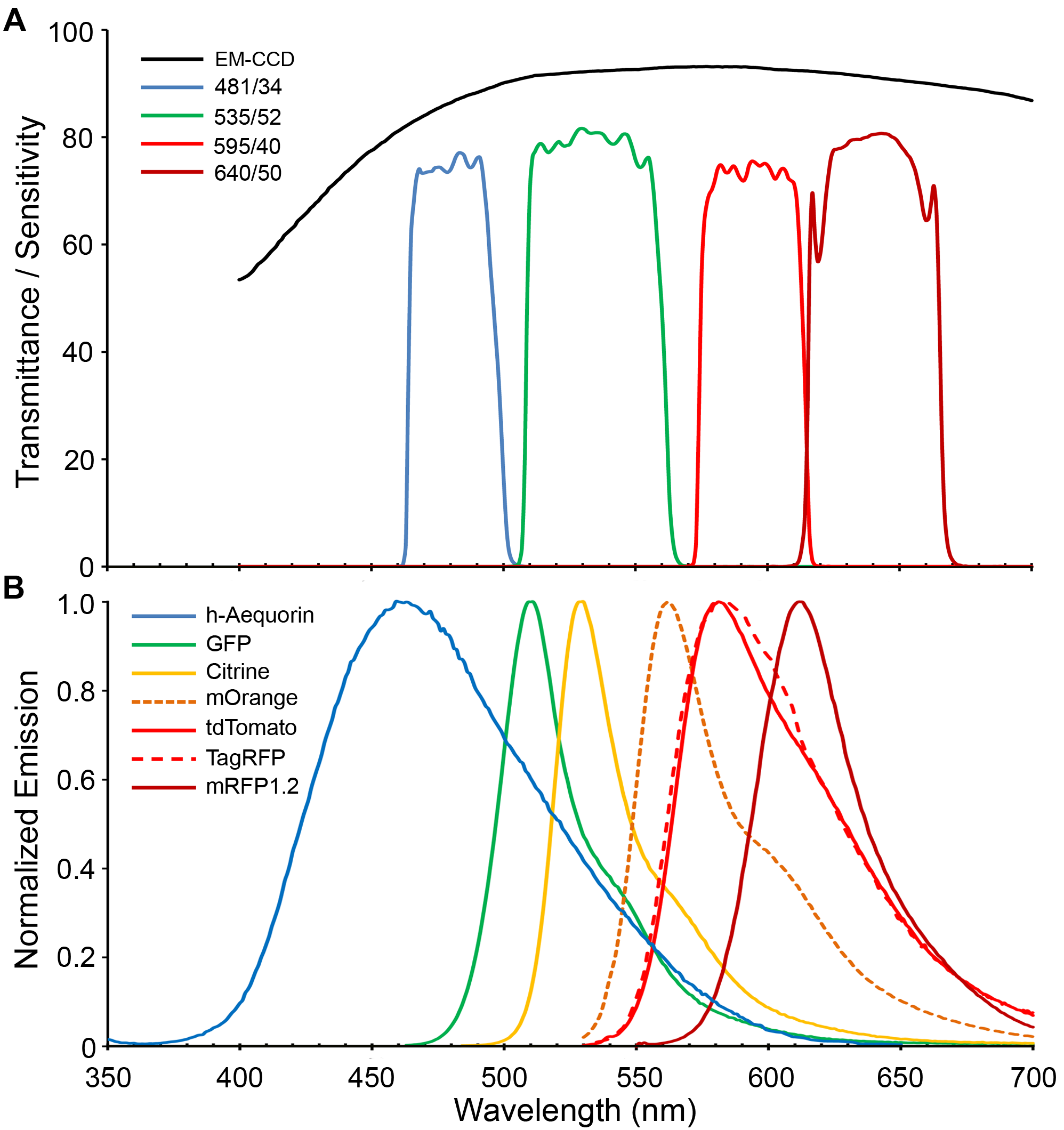

Supplement: Figure S2 — Emission filters and emission spectra of aequorin and various FPs. (A) Transmittance profile of the bandpass interference filters and EM-CCD sensitivity used for characterizing FP-aequorin fusions in HeLa cells and mice. Filters are defined as the center wavelength and bandwidth at half-maximal transmittance. (B) Normalized emission spectra of h-aequorin and various acceptor FPs. The h-Aequorin, GFP, mRFP1.2 and TagRFP data were taken from references [22], [23], [31], [32]. (TIF) [file pone.0019520.s002.tif]

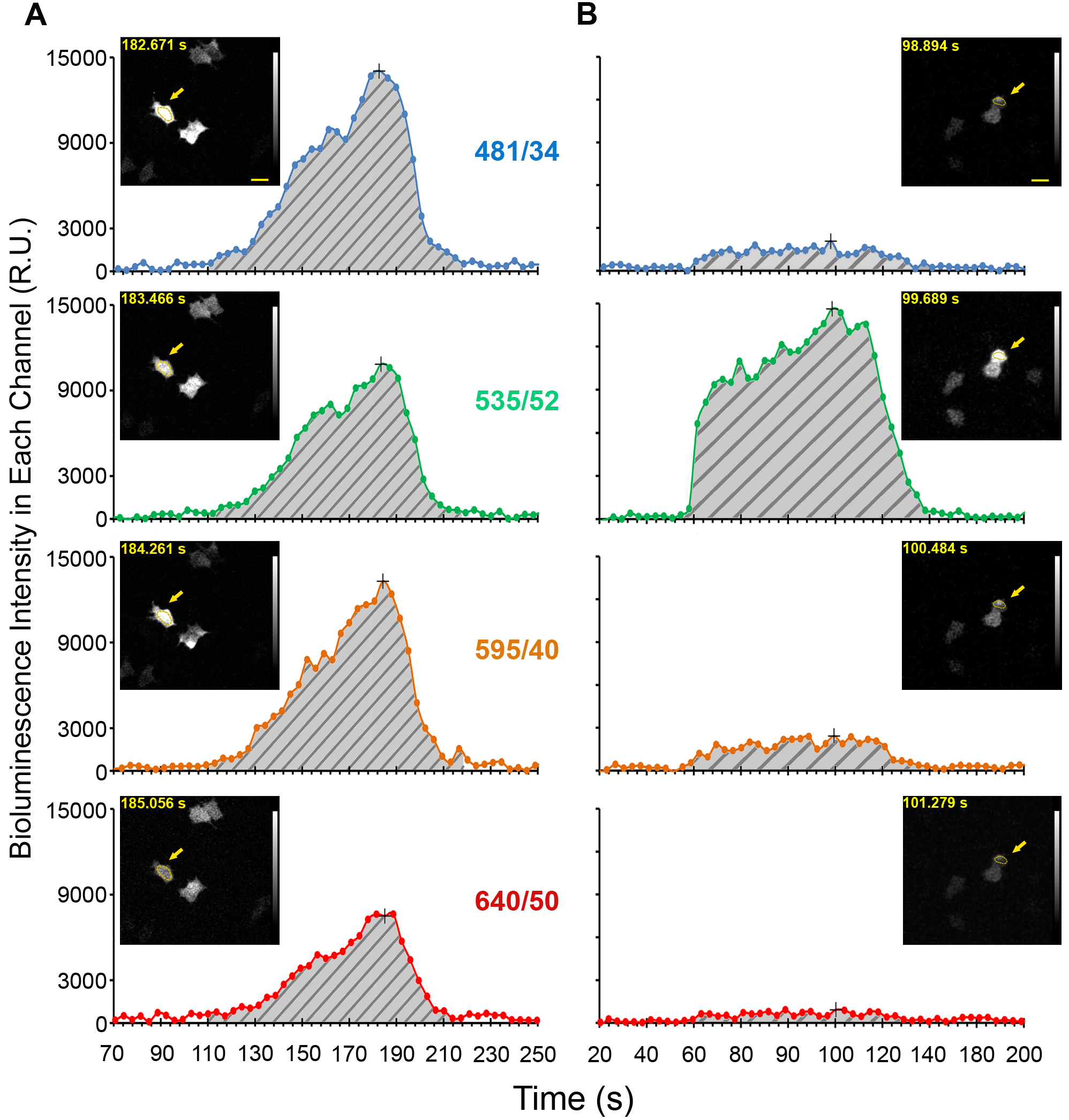

Supplement: Figure S3 — Graphical data analysis of bioluminescence spectral contribution of tdTA and CitA in HeLa cells using the four-channel imaging approach. (A) tdTA (B) CitA. The Ca2+ response of the HeLa cells expressing either chimera during cell permeabilization with saponin was imaged in the four channels in sequential order: a 481-nm channel image was followed 0.8 s later by a 535-nm image, then 595-nm and finally 640-nm images. This cycle was repeated continuously during the experiment. The shape of the Ca2+ response was similar in the four channels. The area under the Ca2+ response curves (gray shading) was integrated for each filter. (TIF) [file pone.0019520.s003.tif]

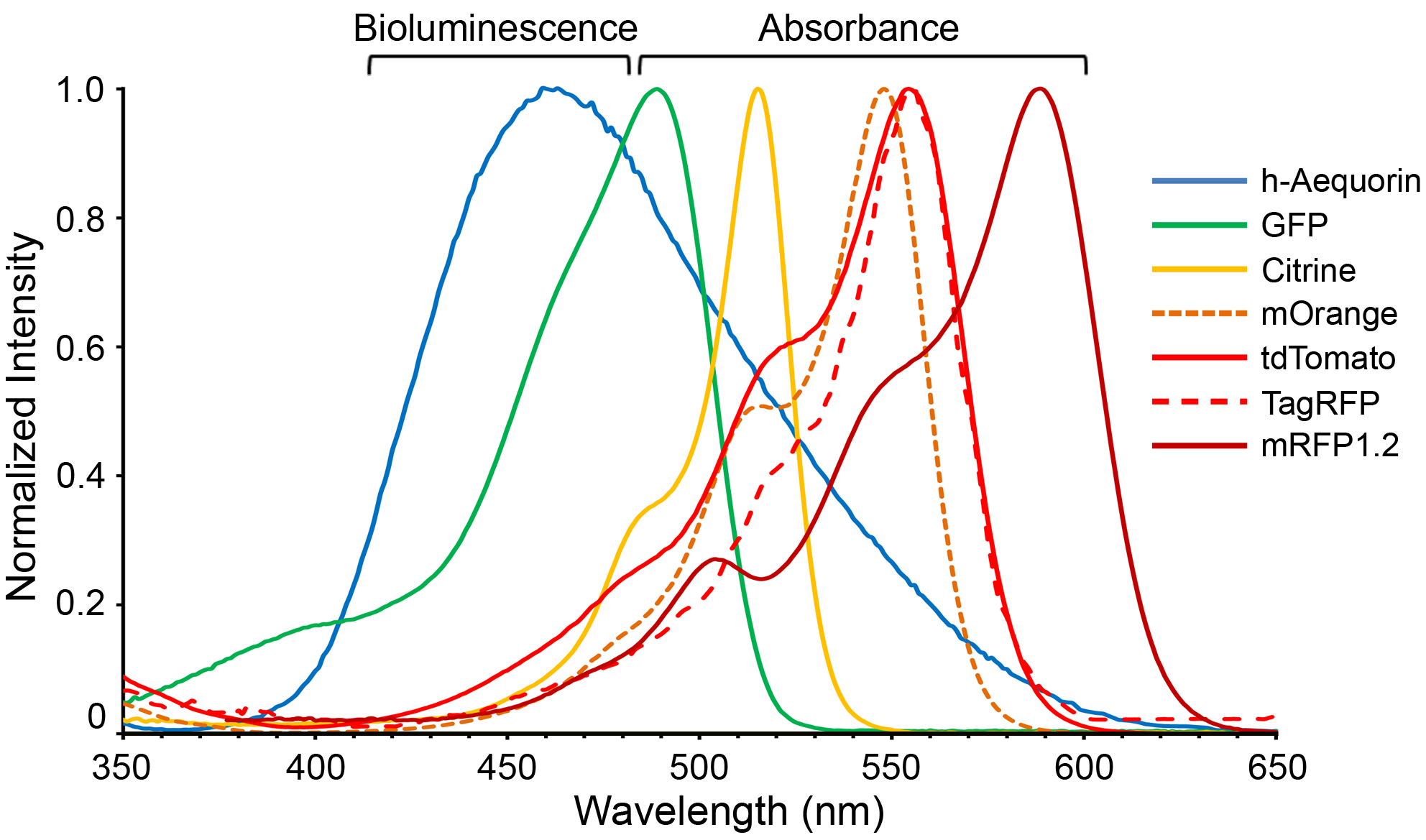

Supplement: Figure S4 — Spectral overlap between h-aequorin bioluminescence and the absorbance spectra of various acceptor FPs. The h-aequorin, GFP, mRFP1.2 and TagRFP data were taken from references [22], [23], [31], [32]. (TIF) [file pone.0019520.s004.tif]

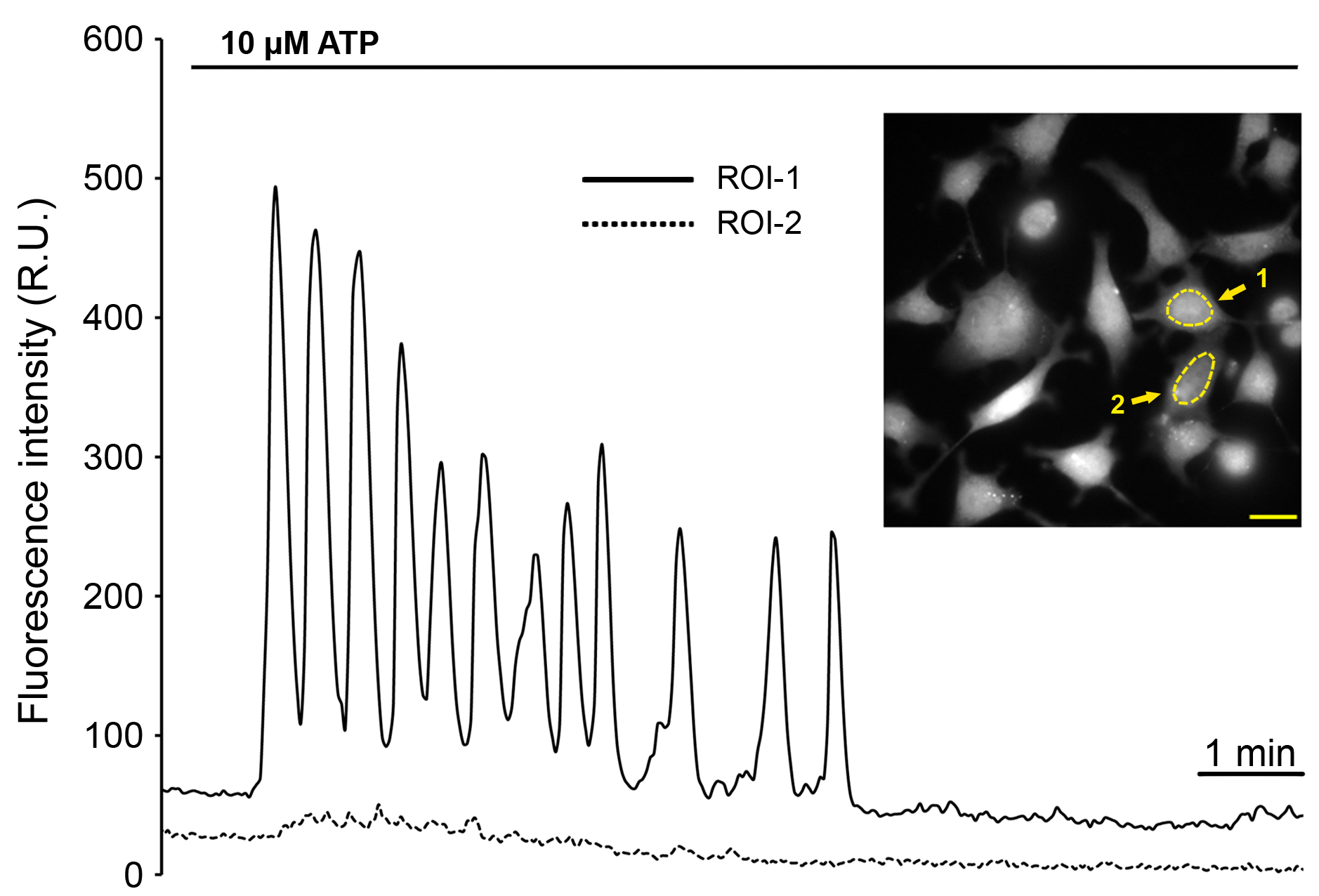

Supplement: Figure S5 — Fluorescence imaging of ATP-induced Ca2+ oscillations in live HeLa cells using Fluo-3. HeLa cells were incubated with 1 µM Fluo3-AM (Molecular Probes) for 45 minutes at room temperature and washed. Image acquisition (500 nm excitation, 122 ms exposure and 2-second image interval using a 40x objective/1.25 NA) started before applying 10 µM ATP in HBSS solution (horizontal bar). ROIs 1 and 2 represent examples of two cells with different Ca2+ responses. Image: 512×512 pixels. Scale bar equals 40 µm. (TIF) [file pone.0019520.s005.tif]
